# Culturally Centered Palliative Care: A Framework for Equitable Neurocritical Care

**DOI:** 10.1007/s12028-024-02041-y

**Published:** 2024-07-02

**Authors:** Paula M. Magee, Tessie W. October

**Affiliations:** 1 Division of Pediatric Critical Care Medicine, Department of Anesthesiology and Critical Care Medicine, Children’s Hospital of Philadelphia, 3401 Civic Center Blvd, 9 Main Suite 9NW45, Philadelphia, PA 19104 USA; 2 Division of Critical Care Medicine, Children’s National Hospital, Washington, DC USA

**Keywords:** Equity, Neurocritical care, Palliative care, Disparities, Racism, Social determinants of health

## Abstract

Health disparities continue to plague racial and ethnic underserved patients in the United States. Disparities extend to the most critically ill patients, including those experiencing neurologic injury and patients at the end of life. Achieving health equity in palliative care in the neurointensive care unit requires clinicians to acknowledge and address structural racism and the social determinants of health. This article highlights racial and ethnic disparities in neurocritical care and palliative care and offers recommendations for an anti-racist approach to palliative care in the neurointensive care unit for clinicians.

## Historical Background of Racial and Ethnic Health Inequities in the United States

Health inequities for racial and ethnic minoritized people in the United States is a public health emergency resulting in significant disparities in outcomes, including among seriously ill patients with neurologic injury who could benefit from palliative care [1, 2]. Our goal as clinicians is to provide high-quality, evidence-based care to all patients. Unfortunately, providing quality clinical care is an insufficient standalone approach to bridge the chasm of health disparities. To achieve health equity, disparities must be understood in the context of structural racism and the social determinants of health (SDOH) [3].

Health behaviors, social and economic factors, and the physical environment are modifiable conditions and environments collectively referred to as SDOH and account for 80–90% of contributors to health outcomes [4, 5]. Racism is a significant driver of differences in SDOH and creates a barrier to achieving health equity because it systematically disadvantages and disempowers racial and ethnic minoritized people.

Mechanistically, racism was and continues to be embedded in US laws and policies that determine access to and the allocation of resources, including health care resources; this is known as structural racism [6].

The impact of structural racism on medicine dates to the nineteenth century and includes the legal, unethical experimentation on enslaved women by Marion Sims [7], the Tuskegee Syphilis Study [8], the experimentation on Black, Puerto Rican, and Asian Soldiers [9], compulsory eugenics [10, 11], and many other injustices in the twentieth century [12]. Concertedly, US policies such as the Hill-Burton Act of 1946, which allowed “separate but equal” health care facilities, operationalized differential care and inequitable access to health care during the Jim Crow era [6]. In clinical practice and research, race was widely considered a biologic construct until 1999 when the Human Genome Project showed no genetic basis for race [13]. As such, decision-making [14] and varying treatment decisions and algorithms [14, 15] inappropriately considered race.

Race is now largely considered a social construct without any biologic influence on health outcomes [16–18]; therefore, clinicians should consider factors such as racism and other SDOH in their approach to addressing health disparities and achieving health equity. Knowing that racial and ethnic minoritized patients will likely experience racism in their health care journey, it is imperative to consider strategies to provide equitable care to our most vulnerable patients—those with serious illness, possibly at the end of life. In this article, we provide a review on disparities in palliative care and neurocritical care and introduce a framework for equitable palliative care in the neurointensive care unit (neuro-ICU).

## Racial Disparities in Neurologic Disease Outcomes

Neurology has not been spared from the prevalence of racial disparities [19]. Black patients have disproportionately higher mortality rates from neurological disorders [20] with a widening Black–White patient mortality gap for neurologic diseases between 2011 and 2019 [21]. Among the 15 most common neurologic diseases, including stroke and other cerebrovascular diseases, Black Americans have the highest age-adjusted mortality rates [21]. To compound the disparity, Black patients have poorer access to neurologic care in general [1]. In neurology and neurocritical care, factors rooted in SDOH and influenced by racism contribute to health disparities, including neighborhood and environmental factors, individual-level factors, structural/societal factors, and health care system factors [19]. In the following section, we highlight disparities in outcomes for patients with stroke and traumatic brain injury (TBI), who are commonly cared for in the neuro-ICU, recognizing that disparities in other neurologic disorders are likely similar.

### Stroke

Black Americans have a higher incidence of ischemic and hemorrhagic stroke when compared with White Americans [19, 22]. For patients experiencing stroke, several studies reported delayed time to initiation of treatment for Black and Mexican American/Hispanic patients. This may be related to emergency medical services (EMS) utilization, with most studies showing lower EMS utilization rates for racially minoritized patients experiencing stroke symptoms [23]. This may also be related to emergency department wait times. One study reported a longer emergency department wait time for Black patients with a longer emergency department to needle time when compared with White patients [23]. In the treatment of stroke, Black and Hispanic patients are less likely to be transferred to endovascular capable centers or to be evaluated by a stroke team when compared with their White counterparts. In addition, there are lower rates of tissue plasminogen activator (tPA) [23, 24] use and thrombectomy in Black and Hispanic patients [23]. Finally, when assessing outcomes, racially minoritized patients have a longer hospital length of stay [24], more ICU days after thrombectomy [25], and increased likelihood of placement of chronic life-sustaining therapies, such as percutaneous gastrostomy tubes following acute ischemic strokes [26].

### TBI

Along the continuum of care from prehospital through rehabilitation, racially and ethnically minoritized patients experience health inequities. Black and Hispanic patients have higher injury severity at presentation [27]. In the acute diagnosis and management of TBI, racially and ethnically minoritized patients experience longer emergency department wait times, have lower triage scores, and have higher rates of leaving the emergency department undiagnosed. Racially and ethnically minoritized patients are also less likely to receive analgesia and different diagnostic procedures and treatments. Hispanic, Asian, and Black veterans are less likely to receive a TBI diagnosis when presenting with positive TBI screens and symptoms [27]. Regarding hospitalization, racially and ethnically minoritized patients have longer lengths of stay; Black patients have a higher odds of experiencing a complication; Black and Hispanic patients with moderate to severe TBI are more likely to have higher in-hospital mortality [27]; and American Indian/Alaska native children and adults have the highest TBI hospitalization and mortality rates [28]. When considering posthospitalization care, Black and Hispanic patients are less likely to receive follow-up care and rehabilitation [27, 28]. When receiving rehabilitation, they are less likely to receive therapies, such as physical therapy, occupational therapy, and speech therapy, and are less likely to receive inpatient rehabilitation, even when adjusting for injury severity [27]. Finally, racially and ethnically minoritized patients are more likely to have poorer psychosocial and functional outcomes after sustaining a TBI [27, 28].

## Racial Disparities in Palliative and End-of-Life Care

**Fig. 1:**
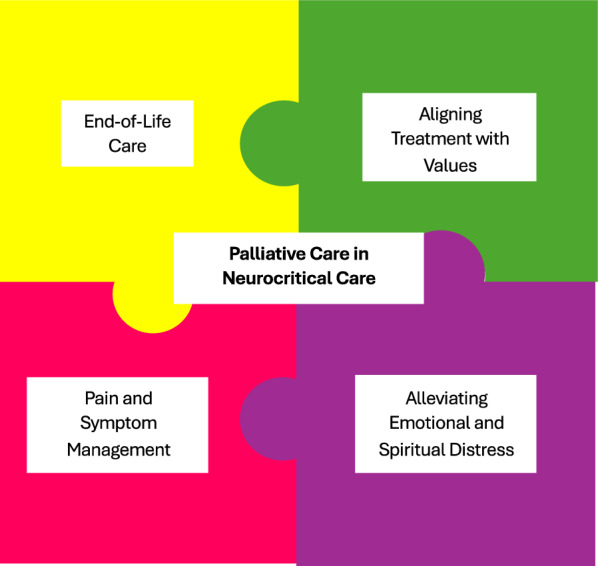
The role of palliative care in neurocritical care

Racial and ethnic disparities extend to palliative care, end-of-life care (EOLC), and hospice [2, 29]. The primary goals of palliative care services include reducing pain and symptoms, aligning treatment with values, alleviating emotional distress, and providing EOLC (Fig. 1). Unfortunately, Black and Hispanic patients are less likely to receive palliative care services, specifically for patients with intracerebral hemorrhage [30] and ischemic stroke [31]. The consequences of not receiving palliative care resources can result in patients living with more pain and suffering, discordant goals, and worse end-of-life experiences [30, 32].

### Pain and Symptom Management

Decades of research have shown that Black and Hispanic patients receive less pain medications compared with White patients [33–36]. Although not specific to neurocritical care, racially and ethnically minoritized patients are more likely to experience lower quality palliative pain care [30], with Black patients feeling their reports of pain are not believed [37]. More specifically, racial and ethnic minority patients receive less comprehensive diagnostic and treatment approaches for pain and are less likely to receive opioid medications for pain across indications and treatment settings [37, 38]. In addition, racially minoritized patients are more likely to be asked about drug use, receive drug testing, and have opioids withheld when controlling for factors such as previous drug use, symptoms on presentation, and diagnosis [33].

### Aligning Treatment with Values

Racially and ethnically minoritized patients experience lower quality of dying and death [39]. Black patients are less likely to receive goals-of-care conversations [30] or be offered an opportunity to complete an advance directive [40]. Even when advance care planning is completed, end-of-life preferences are documented closer to the patient’s death [40] and are less likely to be honored at the end of life [41]. When Black patients express a preference for life-extending EOLC, their preferences are three times less likely to be honored than White patients. Patient preference is also not honored when a do-not-resuscitate order is in place [41, 42]. Similarly, in assessing family conference documentation, clinicians are more likely to recommend withdrawal of life-sustaining therapies for racial and ethnic minority patients [43].

### Alleviating Emotional Distress

Conversations with patients and families with serious neurologic injuries are filled with emotions. Families are grappling with the loss of their loved one as they knew them if they can no longer speak, or have dementia-related memory issues or emotional dysregulation. Clinicians need to be able to recognize these emotions and respond with empathy. Studies show that Black patients are less likely to reveal spontaneous emotions [44] and when they do, clinicians are less likely to address emotional issues or respond with empathy [45, 46].

In addition to emotional support, spiritual support is essential to holistic palliative care. Patients often turn to religion when coping with serious illness [47]. For Black patients, a lack of trust in a health care system that has been untrustworthy increases the reliance on spirituality to guide decision-making [48]. The church is often viewed as a trusted source for health care knowledge, support, and guidance [48, 49], yet clinicians refrain from discussing spirituality with patients [50]. This failure to engage in spiritual discussions leaves patients and families feeling unsupported and further erodes trust.

### EOLC

Life-prolonging therapies and dying with full support are associated with lower quality of dying and death [39], yet racial and ethnic minority patients are more likely to prefer life-sustaining therapies [29, 40]. This disparity may be due to preferences, but it also may be impacted by racial and ethnic minority patients’ access to palliative and hospice services. Racially and ethnically minoritized patients are more likely to have a delay in hospice referral [30] and are less likely to use hospice benefits [51]. Black and Hispanic patients are less likely to die at home or in a nursing home rather than in the hospital when compared with their White counterparts [40]. When considering overall hospital utilization at the end of life, Black patients are more likely to have multiple emergency department visits and hospitalizations and undergo intensive treatment in the last 6 months of life when compared with White decedents [52]. Black and Hispanic patients are also more likely to be exposed to the use of life support or use of dialysis before death compared with White patients [40].

## Palliative Care in the Neuro-ICU

Studies show an association between early integration of palliative care into the ICU and improved outcomes, yet there are limited data on best practices for integrating palliative care into the neuro-ICU [53]. Given the difficulty with early prognostication in the setting of neurologic injury and decreased capacity-making of patients experiencing neurologic injury, early integration of palliative care may be helpful [53, 54]. A few studies identify and highlight palliative care needs or indications in the neuro-ICU, including (1) family emotional support and (2) setting goals of care [53, 55, 56]. Recommendations from the Improving Palliative Care in the ICU Project Advisory Board and the Center to Advance Palliative Care provided a framework to aid clinicians in addressing decision-making around goals of care. The framework includes input from the clinician, with consideration of prognosis using prognostic scores and individual patient factors, and patient/family input, which centers the patient’s values, preferences, and advance directive [54].

Sadly, the literature shows us that racially and ethnically minoritized patients face challenges with engagement of palliative care, and with disparities in access, uptake, and implementation of palliative care services. We also know these same patients are more likely to experience disparities in care after sustaining serious neurologic injury. The intersection of increased need and decreased interface with palliative care services heightens the need for clinicians to provide culturally sensitive, equitable care to these vulnerable patients. In the next section we offer recommendations for a multifaceted anti-racist approach to achieve health equity. These are recommendations clinicians can implement with all patients but are particularly important for historically marginalized patients for whom dignity and respect are often denied during their health care journey.

## An Anti-racist Approach to Palliative Care in the Neuro-ICU

Anti-racism is an active approach that seeks to identify and eliminate racist systems and practices [1]. Applying an anti-racist approach to clinical practice in the neuro-ICU is the responsibility of all clinicians practicing in the neuro-ICU. We know bias and racism exist in each of these components as, described previously. In the following section, we offer strategies clinicians can use to meet the goals of palliative care using an anti-racist approach (Table 1).

**Table 1:** A roadmap for an anti-racist approach to palliative care in the neurointensive care unit

Actionable approach		Associated tenet of palliative care
Affirm narratives	Acknowledge the impact of racism and its contributions to medical mistrust Use high-quality communication skills (actively listen, ask open-ended questions, provide empathetic responses)	Reduce pain and symptoms Align treatment with values Alleviate emotional distress
Lead with empathy and dignity	Restore dignity Make eye contact Use open body language	Alleviate emotional distress
Honor values	Assess the patient’s values and goals Align treatment plans with the patient’s values and goals Engage family early in decision-making	Reduce pain and symptoms Alleviate emotional distress Align treatment with values Provide end-of-life care
Practice cultural humility	Acknowledge biases Self-reflect and be aware of your own identity and how it may differ from others’	Provide end-of-life care Alleviate emotional distress

### Affirm Narratives

An overwhelming body of literature confirms the pervasive pattern of dismissal of racially and ethnically minoritized patient’s concerns when they report pain or share their story with clinicians [57–61]. In addition, racially and ethnically minoritized patients report worse communication with their physicians [62]. Paralleling this data, for patients with severe acute brain injury, family members of racially and ethnically minoritized patients have a higher odds ratio of prognostic discordance with their physician [63].

Patient–clinician communication impacts trust, adherence, disease outcomes, and mortality [64]. To improve outcomes, clinicians must invest in the patient–clinician relationship [65]. This begins with acknowledging the impact of racism and its contributions to medical mistrust for racially and ethnically minoritized patients. To accomplish this feat without causing harm, clinicians should employ high-quality communication skills, in which active listening takes center stage. Patients and families need to feel heard and believed. Clinicians should ask open-ended questions, provide space for emotions, and offer empathetic responses. These important communication skills can be learned, and compassionate clinicians must invest in their professional development to strengthen these skills [66]. Patients remember what we say and how we make them feel. It is our job to listen to their truth and empathize with their experience.

### Lead with Empathy and Dignity

Black patients have experienced significant indignities within the medical community, including medical exploitation for research and medical maltreatment. Furthermore, they experience microaggressions in their day-to-day life. Minoritized patients have described clinicians not willing to touch them or to learn about them as people. Restoring dignity can be an important step in lessening the normalized disrespect minoritized patients experience. Restoring dignity can include greeting Black patients by saying “Good morning,” asking permission to engage and touch, and using surnames when addressing minoritized patients [2]. In addition, clinicians should make eye contact and use open body language; these nonverbal cues can aid in establishing rapport [67] and improve the interpretation of verbal communication [59].

### Honor Values

A larger percentage of Black and Hispanic patients report never having their preferences considered by health care clinicians when compared with White adults [68]. Clinicians not only need to assess the patient’s values and preferences, but they should incorporate these dynamics into their treatment plan and include the patient’s family early in the decision-making [65]. The desire for more therapies at the end of life is rooted in mistrust in a system that has withheld treatments from minoritized patients. Minoritized patients carry that weight with them and need trusting caregivers who truly see them and listen to their story to be able to agree to less therapies. This means that clinicians may need to support life-sustaining therapies if it aligns with the patient’s overall goals, even if they do not align with our own values and preferences as clinicians.

### Practice Cultural Humility

As humans, our experiences, culture, and background shape the lens in which we navigate the world. Practicing with cultural humility acknowledges that our patients also have a complex background that shapes their approach to medical decision-making, which may differ from our own identity. Cultural humility is an iterative process that requires continual refinement and an exploration and awareness of each patient’s lived experience. It also includes recognizing how racism and its sequalae shape individual preferences [37]. Employing cultural humility requires empathy, active listening, acknowledgment of biases, and self-reflection and awareness. To achieve equity in palliative care in the neuro-ICU, clinicians must embrace cultural humility in their approach to patient care, especially when assessing the values of patients and aligning a care plan with their goals and preferences.

## Conclusions

Evidence documents disparities in neurologic outcomes and in palliative care and EOLC for racially and ethnically minoritized patients. Practicing medicine without an appreciation for and an active attempt to dismantle the systems that lead to disparate health outcomes can no longer be considered providing quality care. Applying an anti-racist approach to caring for the most clinically and socially vulnerable patients experiencing serious neurologic illness acknowledges the pervasive nature of racism in medicine and brings us closer to achieving equity.
